# Whole-Genome Sequencing and Bioinformatic Analysis of Isolates from Foodborne Illness Outbreaks of Campylobacter jejuni and Salmonella enterica

**DOI:** 10.1128/JCM.00161-18

**Published:** 2018-10-25

**Authors:** Kelly F. Oakeson, Jennifer Marie Wagner, Andreas Rohrwasser, Robyn Atkinson-Dunn

**Affiliations:** a Utah Department of Health, Utah Public Health Laboratory, Salt Lake City, Utah, USA; Medical College of Wisconsin

**Keywords:** next-generation sequencing, bioinformatics, food-borne pathogens

## Abstract

Whole-genome sequencing (WGS) via next-generation sequencing (NGS) technologies is a powerful tool for determining the relatedness of bacterial isolates in foodborne illness detection and outbreak investigations. WGS has been applied to national outbreaks (for example, Listeria monocytogenes); however, WGS has rarely been used in smaller local outbreaks. The current study demonstrates the superior resolution of genetic and evolutionary relatedness generated by WGS data analysis, compared to pulsed-field gel electrophoresis (PFGE). The current study retrospectively applies WGS and a reference-free bioinformatic analysis to a Utah-specific outbreak of Campylobacter jejuni associated with raw milk and to a national multistate outbreak of Salmonella enterica subsp. enterica serovar Typhimurium associated with rotisserie chicken, both of which were characterized previously by PFGE. Together, these analyses demonstrate how a reference-free WGS workflow is not reliant on determination of a reference sequence, like WGS workflows that are based on single-nucleotide polymorphisms, or the need for curated allele databases, like multilocus sequence typing workflows.

## Abstract (precis)

Whole-genome sequencing (WGS) via next-generation sequencing (NGS) technologies is a powerful tool for determining the relatedness of bacterial isolates in foodborne illness detection and outbreak investigations. WGS has been applied to national outbreaks (for example, Listeria monocytogenes); however, WGS has rarely been used in smaller local outbreaks.

## INTRODUCTION

For the past 20 years, pulsed-field gel electrophoresis (PFGE) has been used almost exclusively for the subtyping of foodborne pathogens isolated from stools and other specimens for surveillance. PFGE has the ability to detect clusters of disease and to aid in the confirmation of the source of outbreaks. Additionally, PFGE standardization allows the results from different laboratories to be compared directly, leading to effective outbreak investigations on both local and national scales (1). PFGE cannot be used to infer phylogenetic relationships, however, and PFGE does not have the power to resolve relationships between unrelated isolates that have identical or nearly identical PFGE banding patterns (2).

The advent of next-generation sequencing (NGS) technologies has greatly reduced the cost, time, and complexity of whole-genome sequencing (WGS). These reductions make it feasible for public health laboratories (PHLs) to implement WGS for bacterial subtyping, virulence determination, antimicrobial resistance determination, and, replacing PFGE, bacterial characterization and comparison. While WGS has the potential to provide results in approximately the same time frame as PFGE, it is reliant on bioinformatic analyses, i.e., computational infrastructure, software workflows, and trained personnel, to obtain these results. These analyses can range in complexity from very basic, i.e., determining the quality of each raw sequence, to very complex, i.e., performing molecular evolutionary analysis of protein-coding genes. The capability to perform bioinformatic analyses is not currently widespread, and many laboratories are not applying WGS to local outbreaks. Nationally, many PHLs contribute raw sequencing reads to the U.S. Centers for Disease Control and Prevention (CDC) for routine surveillance of enteric pathogens and for potential use in large national outbreaks as part of the CDC PulseNet program (3, 4). However, timely analysis of WGS data at the local level is crucial to identify linked cases of illness, which can be an early indication of an outbreak, and to provide phylogenetic and evolutionary relationship information, which can give epidemiological context to an ongoing investigation. This analysis becomes critical as PulseNet moves closer to ending the use of PFGE. WGS can also definitively rule out relationships that might otherwise be associated with a PFGE-defined cluster, saving resources and preventing public health actions that are not necessary.

It is estimated that 3% of the U.S. population drinks raw milk, in part because of perceived health benefits (5, 6). Along with the perceived health benefits comes the risk of ingesting pathogenic bacteria, including Salmonella spp., Campylobacter spp., Shiga toxin-producing Escherichia coli (STEC), and Listeria spp. (7–10). In May 2014, the Utah Public Health Laboratory (UPHL) notified the Utah Department of Health (UDOH) of 3 laboratory-confirmed isolates of Campylobacter jejuni with identical PFGE patterns. All 3 isolates were from patients who reported consumption of raw milk from a dairy in Weber County, Utah. Additional cases were identified in May and June and UDOH opened an investigation on 10 June 2014. Between 9 May and 6 November 2014, 99 cases of C. jejuni infections were identified (11).

The retrospective use of WGS and a reference-free bioinformatic analysis pipeline (12) was applied to this local outbreak of Campylobacter jejuni. Additionally, the same bioinformatic analysis pipeline was applied to a large, diverse, national outbreak of Salmonella enterica subsp. enterica serovar Typhimurium associated with rotisserie chicken. This bioinformatic analysis pipeline is unique, compared to other reference-based bioinformatic analysis pipelines reported by the CDC or the Food and Drug Administration (13, 14), because it is not reliant on a reference genome sequence or curated database.

## MATERIALS AND METHODS

### Campylobacter jejuni sample collection and isolation.

Stools collected by local health departments from persons presumed to have Campylobacter jejuni were plated on Campy CVA agar plates (Thermo Fisher Scientific, Waltham, MA). Isolates previously identified and submitted by clinical laboratories were plated on trypticase soy agar plates supplemented with 5% sheep blood. Agar plates were incubated overnight at 42°C under microaerophilic conditions created in a 2.5-liter jar with 5% O_2_, 10% CO_2_, and 85% N_2_ produced by an Oxoid CampyGen sachet (Thermo Fisher Scientific). The plates were examined at 24, 48, and 72 h for characteristic Campylobacter growth. Colonies exhibiting typical Campylobacter morphology were tested biochemically, with Campylobacter jejuni being identified based on positive oxidase, catalase, and hippurate reactions (15). Isolated colonies were archived at −80°C in a cryopreservative broth containing approximately 20 to 30 individual CryoBeads (Hardy Diagnostics, Santa Maria, CA).

### Campylobacter jejuni PFGE.

PFGE was performed following the method described by Ribot et al. (12), with slight modifications. PFGE plugs were prepared by using a sterile phosphate-buffered saline (PBS)-moistened cotton swab to remove colonies from an agar plate containing fresh (less than 24 h) Campylobacter growth. Colonies were suspended in 2 to 3 ml of PBS, to an optical density measured at a wavelength of 600 nm of 0.48 to 0.52. Next, 400-μl aliquots of the adjusted cell suspensions were transferred to labeled 1.5-ml microcentrifuge tubes containing 20 μl of proteinase K (20 mg/ml stock; Qiagen, Valencia, CA). Then, 400 μl of melted 1% SeaKem Gold agarose (Lonza, Basel, Switzerland) in Tris-EDTA (TE) buffer (10 mM Tris, 1 mM EDTA [pH 8.0]) was added to the cell suspension; the preparation was mixed by pipetting and dispensed into the appropriate wells of the plug mold. The plugs were kept at room temperature for 15 min or at 4°C for 5 min to solidify. Once the plugs had cooled and solidified, they were added to their labeled 50-ml polypropylene screw-cap tubes containing 5 ml of cell lysis buffer (50 mM Tris, 50 mM EDTA [pH 8.0], with 1% Sarcosyl) and 25 μl proteinase K stock solution. Lysis occurred in a 54°C shaking water bath for at least 15 min, with constant vigorous agitation (190 rpm). Plugs were washed a total of six times, i.e., twice with 10 ml of 54°C sterile reagent-grade water and four times with 10 ml of 54°C sterile TE buffer. Washes were performed in a 54°C shaking water bath for at least 10 min, with vigorous shaking. After the final wash, the plugs were placed in labeled 5-ml conical tubes containing 3 ml sterile TE buffer and were stored at 4°C. Approximately 2-mm-wide slices were cut from each Campylobacter plug, using a razor blade, and transferred to a labeled 1.5-ml microcentrifuge tube containing 200 μl of the restriction enzyme mixture, containing 40 U of SmaI or KpnI (Roche Diagnostics Corp., Indianapolis, IN). The plug slices were digested for at least 30 min at 25°C. After digestion, the enzyme mixture was removed and replaced with 200 μl of 0.5× Tris-borate-EDTA (TBE) buffer (10× TBE buffer contains 0.89 M Tris borate and 0.02 M EDTA [pH 8.3]), and the plug slices were allowed to stand at room temperature for 5 min. The digested plug slices were loaded onto a comb, and 1% SeaKem Gold agarose in 0.5× TBE buffer was poured into the gel form containing the comb. After 30 min, the comb and the frame of the gel form were removed, and the gel was placed in a CHEF Mapper electrophoresis system (Bio-Rad, Hercules, CA). Gels were run with an initial switch time of 6.76 s, which was increased linearly to reach a final switch time of 35.38 s. Gels were subjected to electrophoresis for approximately 19 h at 6 V/cm, in 0.5× TBE running buffer, at 14°C. Gels were stained with ethidium bromide (Amresco) and destained in water. Pattern visualization was obtained under UV transillumination aided by a Gel Doc XR gel analysis system (Bio-Rad). The PFGE patterns were analyzed using BioNumerics v6.6 11 (Applied Maths, Austin, TX).

### Campylobacter jejuni DNA isolation.

Frozen isolates were removed from the −80°C freezer and allowed to thaw briefly. A sterile wooden stick was then used to remove approximately 5 CryoBeads (Thermo Fisher Scientific) from the freezer vial, and they were plated on a blood agar plate (Thermo Fisher Scientific) with gentle rolling. Plates were incubated overnight at 42°C under microaerophilic conditions created in a 2.5-liter jar with 5% O_2_, 10% CO_2_, and 85% N_2_ produced by an Oxoid CampyGen sachet (Thermo Fisher Scientific). Using a sterile loop, approximately 1 μl of growth was transferred to 1 ml of sterile PBS and vortex-mixed. These cells were centrifuged at 5,000 × *g* for 10 min. The supernatant was removed, and the pelleted bacteria were used for DNA extraction using the DNeasy blood and tissue kit (Qiagen). For isolates that could not be recovered from frozen cultures, DNA was extracted directly from the PFGE plugs with the QIAquick gel extraction kit (Qiagen). DNA in the final eluate was quantified by using a Qubit 3.0 fluorometer (Thermo Fisher Scientific).

### Campylobacter jejuni sequencing library construction and sequencing.

Total genomic DNA from each isolate was used to construct a paired-end sequencing library using the Illumina Nextera XT DNA library preparation kit (Illumina, San Diego, CA). Individual libraries were multiplexed, and 20 samples per run were sequenced on the Illumina MiSeq platform (Illumina), using the MiSeq reagent kit v2 (Illumina); this generated an average of 1,699,697 paired-end reads of 250 bases in length.

### Salmonella enterica subsp. enterica serovar Typhimurium sequencing.

Paired-end sequencing reads for S. enterica were downloaded from the NCBI Sequence Read Archive (SRA) and processed through the bioinformatic analysis pipeline described below. NCBI accession numbers were obtained from the CDC for samples associated with a multistate outbreak of S. enterica (outbreak code 1609WAJPX-1).

### Bioinformatic analysis pipeline.

Bioinformatic analysis was performed using a previously described pipeline (16), as briefly described below.

### (i) Paired-end read quality control.

All paired-end reads were quality filtered with Trimmomatic v0.36 (17). Low-quality paired-end reads (Phred scores of <30) and reads shorter than 50 bp were discarded. Technical sequences, including partial adapter sequences, were trimmed from the paired-end reads.

### (ii) Genome assembly and annotation.

Quality-controlled paired-end reads from each isolate were *de novo* assembled independently using the SPAdes v3.9.1 assembler (18, 19), using the “careful” option in order to reduce the number of mismatches and short indels in the final assembly. QUAST (20) was used to generate basic statistics, including the total number of contigs, contig length, GC proportion, and *N*_50_, for each genome assembly and to quickly evaluate the quality of each draft assembly. The assembled draft genome sequence of each isolate was annotated with Prokka v1.12-beta (21), with the “compliant” setting limiting the minimum contig length that was annotated to >200 bp.

### (iii) Phylogenetic analysis.

The pan-genome of the assembled and annotated draft genome sequences was generated by Roary v3.7.0 (22). Four additional genomes (GenBank accession numbers GCF_000254855.1, GCF_000251165.1, GCF_000835305.1, and GCF_000009085.1) were included for phylogenetic analysis of Campylobacter jejuni, and 2 additional genomes (GenBank accession numbers GCF_000784245.1 and GCF_000195995.1) were used for the analysis of Salmonella enterica subsp. enterica serovar Typhimurium. These additional genomes were included in the pan-genome to provide appropriate resolution within the data set and to provide an outgroup to properly root the phylogenetic trees. The pan-genome, excluding repetitive sequences, insertion sequence elements, and any mobile genetic elements, was then used to generate a nucleotide alignment of all shared homologous genes concatenated together. The alignments were manually inspected for misalignments and are available from K.F.O. upon request. JModelTest (23) was then used to infer the most appropriate model of sequence evolution (general time reversible plus invariable site plus discrete gamma [GTR+I+G] model) for subsequent analyses. Analysis of pan-genome concatenated homologous gene sequences was performed using the maximum likelihood approach implemented in RAxML (24), with 1,000 rapid bootstrap replicates. The resulting phylogenetic trees were then visualized with TreeView2 (25) and decorated using Adobe Illustrator (Adobe, San Jose, CA). Nucleotide diversity calculations were performed using the nucleotide alignments generated by Roary with the Pegas package (26), as implemented in R Studio (RStudio Inc., Boston, MA), with default parameters.

### Accession number(s).

Campylobacter jejuni and Salmonella enterica subsp. enterica serovar Typhimurium sequencing reads are available in the NCBI SRA database. A list of SRA accession numbers used in this work is available in Table S1 in the supplemental material. The SRA accession numbers for the newly determined Campylobacter jejuni sequences are SRS1929564 to SRS1929567, SRS1929569, SRS1929570, SRS1929573 to SRS1929576, SRS1929580, SRS1929582, SRS1929585, SRS1929587, SRS1929589, SRS1929590, SRS1929593, SRS1929594, SRS1929597, SRS1929599 to SRS1929602, SRS1929605, SRS1929607, SRS1929610 to SRS1929612, SRS1929615 to SRS1929617, SRS1929619, SRS1929620, SRS1929624, SRS1929625, SRS1929627, SRS1929629, SRS1929630, SRS1929632, SRS1946013 to SRS1946026, SRS1946030, SRS1946031, SRS1946033, SRS1946037, SRS1946040, SRS1948189 to SRS1948207, and SRS2012998 (see Table S1).

## RESULTS

### Campylobacter jejuni PFGE.

**FIG 1:**
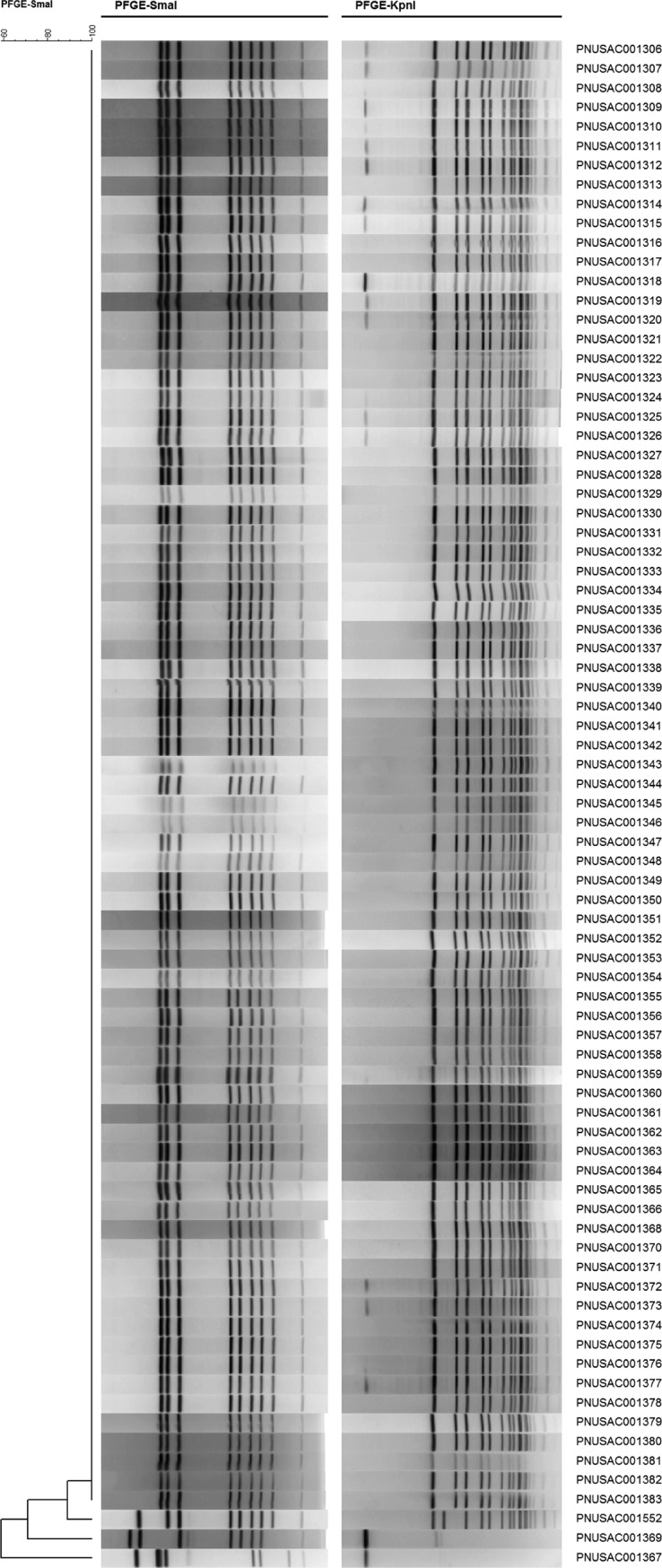
PFGE results for C. jejuni isolates associated with the outbreak. First and second enzyme digestion patterns (SmaI and KpnI, respectively) are shown.

PFGE analysis of 79 isolates associated with the 99 identified Campylobacter jejuni cases (61 clinical isolates and 18 environmental isolates) related to a raw milk outbreak revealed that 75 isolates, including 16 environmental isolates, shared identical PFGE profiles for both SmaI and KpnI enzyme digestions, designated UTDBDS16.944 and UTDBDK02.053, respectively (Fig. 1). A single clinical isolate, PNUSAC001324, had a different KpnI enzyme profile, designated UTDBDK02.044, but shared the same SmaI enzyme pattern with the other 75 isolates. Two environmental isolates, PNSUAC001367 and PNSAUC001369, had different SmaI enzyme PFGE profiles but identical KpnI enzyme profiles and are presented as outgroups to the main outbreak in Fig. 1. The clinical isolate PNUSAC001552 was unique for both SmaI and KpnI enzyme profiles and was initially excluded; however, epidemiological data implicated this case in the outbreak (11).

### Campylobacter jejuni draft genome assemblies, annotations, and phylogenetic analysis.

A total of 79 isolates recovered from the 99 identified Campylobacter jejuni cases (61 clinical isolates and 18 environmental isolates) were sequenced, *de novo* assembled, and annotated (Fig. 1). The average *N*_50_ of the draft assemblies was 273,070, with an average number of >500-bp scaffolds of 26, and the average total length of >500-bp scaffolds was 1.68 Mb, identical to the median length of Campylobacter jejuni genomes in the NCBI database (see Table S2 in the supplemental material). These assembly statistics indicated that all of the sequenced isolates yielded high-quality assemblies that contained nearly complete genomic contents and gene inventories and could be used for pan-genome analysis and phylogenetic analysis (20). The clinical isolate PNUSAC001324 contained contamination from Parabacteroides distasonis, a common member of normal mammalian gastrointestinal flora. The contamination reads were removed computationally by aligning all quality control paired-end reads to the *P*. distasonis genome and discarding any reads that aligned unambiguously. The remaining paired-end reads were then used as input for the assembly, and the resulting assembly was manually screened for any contigs containing *P*. distasonis sequences. The genome assembly statistics for the genome were comparable to those for other C. jejuni assemblies (Table S2). Additional contamination screening was performed for all sequence reads, to identify and to remove any potential contamination (including human and other eukaryote contamination), and no contamination was found.

**FIG 2:**
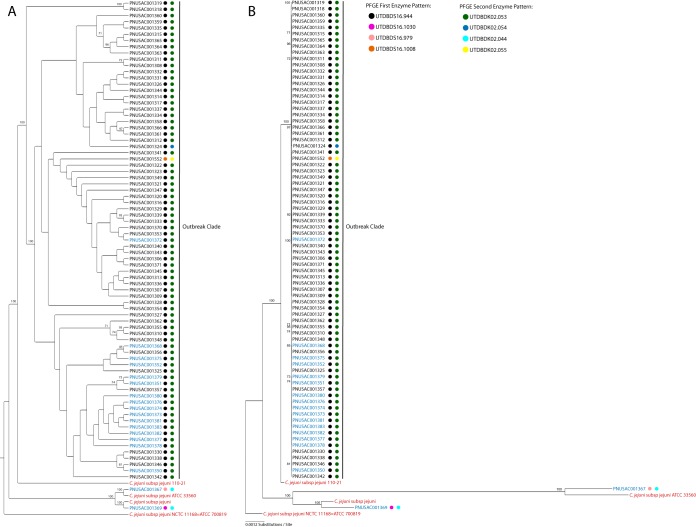
Maximum likelihood trees for 1,223 concatenated protein-coding genes. Labels in blue indicate isolates from raw milk bulk storage tanks, labels in black indicate isolates from patients, and labels in red indicate references strains used to root the tree. Colored squares next to the accession numbers represent first and second enzyme digestion PFGE banding patterns. (A) Cladogram representation of the maximum likelihood tree, with bootstrap support values above 70 shown. (B) Phylogram representation of the maximum likelihood tree, with branch lengths proportional to the number of nucleotide substitutions per site.

Phylogenetic analysis of 1,223 concatenated, shared, homologous, protein-coding genes placed 77 of the 79 isolates, including isolate PNUSAC001552, which had unique PFGE profiles and was originally excluded as part of the laboratory-defined cluster, in a single well-supported clade (Fig. 2). Isolates PNSUAC001367 and PNSAUC001369, which also had differing PFGE profiles, were placed in separate clades, with long-branch lengths indicating that they were phylogenetically dissimilar from the main outbreak clade. Isolates PNSUAC001367 and PNSAUC001369 were obtained from raw milk storage tanks and represented strains of C. jejuni that were present in the raw milk but were not detected in infected patients. Additionally, all patient-derived isolates were phylogenetically distantly related to these 2 isolates. Aside from the placement of PNUSAC001552 in the main outbreak clade, the phylogenetic analysis was in close agreement with the PFGE results (11).

Nucleotide diversity calculations add additional support to the phylogenetic analysis (27); the nucleotide diversity of the outbreak clade was 5.925e−6, compared to greater nucleotide diversity of the entire tree of 0.00121 and even greater diversity of the non-outbreak-related isolates of 0.0126. The low nucleotide diversity of the outbreak clade again indicates the high level of relatedness of these isolates.

### Salmonella enterica subsp. enterica serovar Typhimurium PFGE.

**FIG 3:**
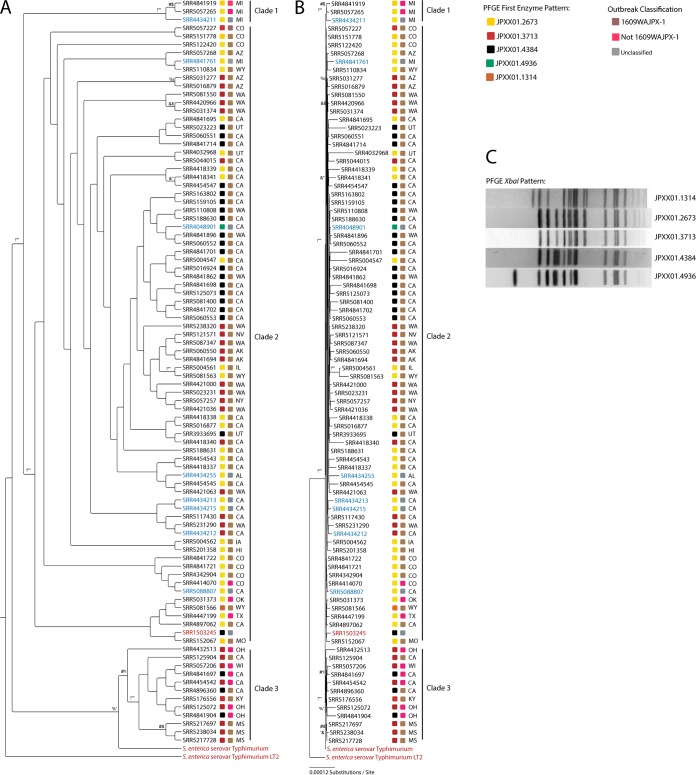
Maximum likelihood trees for 4,037 concatenated protein-coding genes. Labels in blue indicate isolates from the USDA Food Safety and Inspection Service, labels in black indicate isolates from patients, and labels in red indicate references strains used to root the tree. Colored squares next to the accession numbers represent the PFGE banding pattern and inclusion or exclusion from outbreak 1609WAJPX-1. (A) Cladogram representation of the maximum likelihood tree, with bootstrap support values above 70 shown. (B) Phylogram representation of the maximum likelihood tree, with branch lengths proportional to the number of nucleotide substitutions per site. (C) PFGE banding patterns associated with CDC outbreak code 1609WAJPX-1.

In July and November 2016, during routine PulseNet PFGE analysis, the UPHL found 3 Salmonella enterica subsp. enterica serovar Typhimurium isolates (SRR3933695, SRR4032968, and SRR5023223) that had banding patterns similar to those of isolates associated with illnesses related to rotisserie chicken. These PFGE banding patterns were associated with CDC outbreak code 1609WAJPX-1. Isolates SRR3933695 and SRR4032968 shared pattern JPXX01.2673, and isolate SRR5023223 had pattern JPXX01.4384 (Fig. 3C).

### Salmonella enterica subsp. enterica serovar Typhimurium draft genome assemblies, annotations, and phylogenetic analysis.

A total of 88 S. enterica isolates (80 clinical isolates and 8 environmental isolates) implicated in a multistate outbreak related to rotisserie chicken (CDC PulseNet, personal communication) were obtained from the NCBI SRA (6), *de novo* assembled, and annotated. The average *N*_50_ of the draft assemblies was 282,732, with an average number of >500-bp scaffolds of 73.64, and the average total length of >500-bp scaffolds was 4.93 Mb (Table S2). These assembly statistics indicated that all of the sequenced isolates yielded high-quality assemblies that contained nearly complete genomic contents and gene inventories and could be used for pan-genome analysis and phylogenic analysis (20).

Phylogenetic analysis of 4,037 concatenated, shared, homologous, protein-coding genes, including 3 reference strains, placed all 88 isolates in 3 well-supported clades (Fig. 3A); however, all isolates, regardless of clade, were on very short branches, indicating close phylogenetic relationships (Fig. 3B). Isolates with the same PFGE banding patterns were spread throughout the tree and, in many cases, isolates from the same state were distributed across the tree, with little to no clustering by state. Clade 1 consisted of 2 patient-derived isolates and 1 environmental isolate. The 3 isolates had identical PFGE patterns, and all originated from Michigan. Clade 2, the main outbreak clade, contained the majority of the patient-derived isolates associated with the outbreak, as well as 7 of the 8 environmental or food-derived isolates. This clade also contained the reference genome assembly generated by the CDC, SRR1503245 (CDC PulseNet, personal communication). Clade 3 consisted entirely of patient-derived isolates, with no environmental or food isolates. This clade did contain all 3 isolates from Mississippi, which was the only instance in the tree in which all isolates from one state were grouped in a single well-supported subclade.

## DISCUSSION

PFGE has been used since the inception of the CDC PulseNet program as the method of choice for bacterial “fingerprinting”; however, PFGE has limitations, compared to WGS. PFGE cannot indicate the phylogenetic relationships of isolates. This shortcoming is evident in the results for the two outbreaks presented here. In both cases, PFGE lacked the resolution to determine the outbreak relatedness of all isolates, whereas the genomic approaches in the current study indicate the phylogenetic relationships of the isolates, as well as providing nearly complete genotypic information for each isolate.

The UPHL performed retrospective WGS and reference-free bioinformatic analysis, including phylogenetic analysis, on 61 clinical isolates of Campylobacter jejuni obtained from confirmed cases associated with a raw milk outbreak and 18 isolates obtained from bulk raw milk storage tanks and packaged raw milk from the suspect dairy display case. Sixty-one of the patient isolates and 14 of the raw milk isolates formed a single clade with extremely short branch lengths, with high statistical support and 100% bootstrap support, indicating close phylogenetic relatedness (28). The PFGE result mirrors the WGS result except for the placement of isolate PNSUAC001552 outside the main outbreak group. Isolate PNSUAC001552 was isolated from a clinical specimen from a patient who had not consumed raw milk but reported contact with a confirmed outbreak patient who had consumed contaminated raw milk. Multiple genetic events, such as point mutations, insertions, and deletions, can lead to the loss or gain of restriction sites, as well as changes in the methylation status of the DNA being digested. This can cause related isolates to have distinct PFGE banding patterns (29). The distinct PFGE banding pattern of isolate PNSUAC001552 might have been the result of one or multiple genetic events. While these mutations might have a large impact on the PFGE pattern, such events would have little impact on determination of the genetic relatedness to other isolates, allowing WGS to correctly include this isolate in the outbreak.

Isolates PNUSAC001367 and PNUSAC001369 were derived from samples taken from raw milk bulk storage tanks. These 2 isolates are located outside the main outbreak clade and on much longer branches, indicating that they are phylogenetically distantly related to the main outbreak clade. These 2 isolates are more closely related to 2 different reference strains of Campylobacter jejuni than to the isolates derived from milk samples. The results of WGS and subsequent bioinformatic analysis correlate well with the PFGE and epidemiological outbreak investigation results, with the exception of 1 isolate that was excluded from the outbreak by PFGE. The concordance of these results indicates that WGS and the analysis applied to this outbreak were able to correctly resolve the relatedness of all of the isolates analyzed, with greater resolution than PFGE.

A limitation of this data set is that, from the 99 cases of Campylobacter jejuni infections identified through laboratory analysis and patient interviews, only 61 isolates were available for characterization. Analysis of the missing isolates would either group them with the main outbreak clade, group them outside the main outbreak clade, or a mixture of the two. The addition of those isolates would not change the underlying fact that phylogenetically related isolates group together, based on shared genetic composition and shared evolutionary history. Incomplete sampling is a limitation of any investigation of foodborne illness, as the vast majority of illnesses go unreported (30) and laboratory techniques for isolation and detection are not 100% sensitive or specific.

To determine how this analysis would perform with a complex data set that includes multiple PFGE patterns spread across multiple states, a multistate outbreak of Salmonella enterica subsp. enterica serovar Typhimurium was analyzed. In July and November 2016, during routine PulseNet PFGE analysis, UPHL found 3 Salmonella enterica isolates that had banding patterns similar to those of isolates associated with illnesses related to rotisserie chicken. In contrast to the single PFGE pattern of the C. jejuni raw milk outbreak, the nationwide S. enterica outbreak consisted of 5 distinct PFGE patterns spread across 20 states. This outbreak involved a total 91 S. enterica genomes in the NCBI database, 89 of which were *de novo* assembled and annotated from publicly available NCBI SRA data (31). The analysis showed that all of the isolates involved in the outbreak were phylogenetically related (Fig. 3B), as indicated by short branch lengths. The scale bar in Fig. 3B represents 0.00012 nucleotide substitutions per site. Only SRR4032968, with the longest branch in the tree, approached this very small number of substitutions. Despite this level of genomic relatedness, the analysis was able to distinguish 3 separate clades with high statistical support, all with bootstrap support above 70%. In this reference-free analysis, clade 1 consisted of 2 patient-derived isolates and 1 food-derived isolate. All 3 isolates had the same PFGE pattern and were included in the initial outbreak but were later removed based on the result of the CDC high-quality single-nucleotide polymorphism (hqSNP) analysis (CDC PulseNet, personal communication). This variation highlights how different bioinformatic analysis workflows can affect the scope of an outbreak. The analysis also indicated that these isolates were distinct from clade 2, the main outbreak clade. Clade 3 also was distinct from clade 2; however, clade 3 contains a mixture of isolates that the CDC has classified as part of the outbreak and isolates that have been ruled out by hqSNP analysis. The inclusion of outbreak-related isolates in clade 3 and the inclusion of 3 non-outbreak-related isolates in the main outbreak clade require further investigation, in order to resolve the conflicts between this study and the CDC hqSNP analysis. This was a retrospective study, however, and the CDC hqSNP trees and analysis were no longer available; the only accessible data were the publicly available data in the NCBI databases, associated metadata, and limited personal communication with the CDC PulseNet program. The isolates that were removed from the CDC outbreak definition through hqSNP analysis but were included with the reference-free method are difficult to address without more specific information from the CDC regarding exactly why the isolates were removed. It is possible that the isolates had more single-nucleotide polymorphisms (SNPs) than the arbitrarily defined SNP cutoff value for inclusion in the outbreak.

Seemingly discrepant results emphasize the power of reference-free analyses. Reference-free analyses do not rely on a judgment of relatedness based on vague values of SNP differences. Instead, any reference-free analysis indicates only the genetic relatedness and shared evolutionary history of the isolates. In this capacity, reference-free analyses provide better unbiased entry points for epidemiologists initiating outbreak analyses.

Foodborne illness outbreaks often are the result of a point source of bacterial contamination, which is then dispersed to individuals who consume the adulterated food. If a point source of contamination was the underlying cause of the nationwide S. enterica outbreak, then isolates would not be expected to cluster based on geographic location in molecular evolutionary analyses; isolates would cluster based on their genetic relatedness and evolutionary history.

The 5 PFGE patterns associated with this outbreak were similar, with only a small number of bands differing between the patterns (Fig. 3C). However, PFGE was not sufficient to resolve the relatedness of isolates in this outbreak. With the use of PFGE alone, the outbreak would have contained all 88 isolates. When the higher-resolution WGS analysis is used, however, more accurate phylogenetic relationships can be determined.

Research has shown that genetic diversity and genomic plasticity in bacteria increase with geographic distance (32–34), which can make it difficult to perform SNP-based analyses (35). As expected, the reference-free analysis and pipeline applied here were not affected by high levels of nucleotide diversity and identified over 4,000 shared, homologous, protein-coding genes, which enabled the construction of a high-resolution phylogenetic tree with high levels of statistical support for several clades. This result indicates that the described analysis and bioinformatic approach can be applied to large multistate outbreaks.

A significant benefit of this bioinformatic analysis is that a reference genome sequence is not needed. This reference-free analysis removes the critical step of selecting the genome sequence of a closely related organism, which can dramatically affect the outcome of an analysis (35). Additionally, the bioinformatic analysis used is not reliant on curated multilocus sequence typing (MLST) databases. This reference-free approach not only allows the resolution of relationships between highly similar bacterial isolates but also allows the analysis of isolates that may not have representation in a MLST database or highly divergent strains that may not have an available reference genome sequence. Additionally, the reference-free analysis does not require the ongoing updating and maintenance of multiple databases. The reference-free analysis allows for the addition of historical sequence data and other known sequences, to provide context to the analysis. The addition of these historical or known sequences allows the surveillance of pathogens associated with foodborne illnesses, the detection of possible outbreak clusters, and the surveillance of outbreaks over long periods and in relation to known sources of contamination.

The genotypic information generated by WGS can be further exploited to gain insights into virulence factors, antimicrobial resistance, and molecular evolution for outbreak-associated isolates (34, 35). The bioinformatic analysis workflow used in this study has the ability to provide nearly complete genotype information for the isolates analyzed. The data are not limited to revealing the presence or absence of virulence factor or antimicrobial resistance genes but can provide insights into the molecular evolutionary forces behind these very important genotypes.

## Supplementary Material

Supplemental file 1

Supplemental file 2
